# Verbal Aggression from Care Recipients as a Risk Factor among Nursing Staff: A Study on Burnout in the JD-R Model Perspective

**DOI:** 10.1155/2015/215267

**Published:** 2015-10-19

**Authors:** Sara Viotti, Silvia Gilardi, Chiara Guglielmetti, Daniela Converso

**Affiliations:** ^1^Dipartimento di Psicologia, Università degli Studi di Torino, Via Verdi 8, 10124 Torino, Italy; ^2^Dipartimento di Scienze Sociali e Politiche, Università degli Studi di Milano, Via del Conservatorio 7, 20122 Milano, Italy; ^3^Dipartimento di Economia, Management e Metodi Quantitativi, Università degli Studi di Milano, Via Conservatorio 7, 20122 Milano, Italy

## Abstract

Among nursing staff, the risk of experiencing violence, especially verbal aggression, is particularly relevant. The present study, developed in the theoretical framework of the Job Demands-Resources model (JD-R), has two main aims: (a) to examine the association between verbal aggression and job burnout in both nurses and nurse's aides and (b) to assess whether job content, social resources, and organizational resources lessen the negative impact of verbal aggression on burnout in the two professional groups. The cross-sectional study uses a dataset that consists of 630 workers (522 nurses and 108 nurse's aides) employed in emergency and medical units. High associations were found between verbal aggression and job burnout in both professional groups. Moderated hierarchical regressions showed that, among nurses, only the job content level resources moderated the effects of the verbal aggression on job burnout. Among nurse's aides, the opposite was found. Some resources on the social and organizational levels but none of the job content level resources buffered the effects of verbal aggression on workers burnout. The study highlights the crucial role of different types of resources in protecting nursing staff from the detrimental effects of verbal aggression on job burnout.

## 1. Introduction

In the workplace, nursing staff are exposed to various factors that are likely to jeopardize their health and safety. Among these, the risk of experiencing violence is particularly relevant. Work-related violence includes both physically and psychologically violent incidents in which staff members are abused, threatened, or assaulted. It can be defined as “any threat, physical, and/or psychological, that is directed toward a person while at work” [1].

More specifically, in the health care sector, the most common violence is the so-called Type II category, described as the following in the Californian Occupational Safety and Health Administration classification [2, 3]: events involving aggressions by someone who is either the recipient of a service provided by the affected workplace or the victim.

Europe is recently witnessing a progressive increase of Type II violence, which is considered an “emerging epidemic” [4, 5]. In a study across 10 European countries, Camerino et al. [6] found that 9.9% of nurses face violence from patients or patients' relatives at least once a week (countries over the European average: France, 19.5%; UK, 12.3%; Germany, 11.5%; and Italy, 10.3%). This violence mainly consists of verbal aggression, including loud and demanding verbal hostility or verbal threats of the intent to do harm [7, 8].

Because a higher level of violence is expected in those units where patients may initiate more verbal or physical threats (i.e., psychiatric wards or elderly patient areas) or where emergencies and workload are massive (i.e., out-of-hours primary care, emergency, and ICU units), most of the research has been conducted in these specific contexts [9–13]. The existing literature mainly highlights the negative effects of violence exposure in terms of physical and psychological health, and it rarely investigates the protective factors or the positive resources that workers possess/adopt to buffer them [14–16].

The present study focused on verbal aggression, which is one of the most common forms of Type II violence incurred by nursing staffs. Using the framework of the Job Demands-Resources model (JD-R [17–19]), the study intends to investigate the relationship between verbal aggression and burnout among two categories within the nursing profession, namely nurses and nurse's aides. The study also examines whether and which different kinds of job resources are able to buffer the impact of verbal aggression on burnout among the two subsamples considered.

Verbal aggression is a form of direct psychological aggression that includes yelling at the service provider or making sarcastic or offensive remarks [20]. According to the JD-R model [17–19], verbal aggression can be considered a job demand because it is a psychological aspect of the job that requires sustained psychological effort and is therefore associated with certain physiological and/or psychological costs. The present paper focuses on a specific psychological cost, namely, job burnout, which, as the literature has extensively highlighted, represents a particularly relevant concern within the nursing profession [21, 22]. According to Green et al. [23], burnout is a syndrome recognizable by two core dimensions: emotional exhaustion, which refers to the depletion of the energy process, and depersonalization, which indicates a highly detached attitude toward patients.

The fact that being exposed to verbal aggression may lead to burnout is suggested by one of the main assumptions of the JD-R model [17–19], the* health impairment hypothesis*. In accordance with this assumption and the Consarvation of Resources theory (COR) [24], verbal aggression may deplete workers' energy, activating a loss cycle that can lead to exhausting employees' mental and physical resources. In addition, as a consequence of perceiving contact with the patient as a threat, the workers may adopt an attitude of avoidance, such as depersonalization. From an empirical point of view, many studies carried out both within the customer service workers' population [25–28] and within the health sector workers' population, in particular [29–34], confirmed the positive association between verbal aggression and burnout. Based on that, in the present study, it is expected to find a significant and positive relationship between verbal aggression and respectively emotional exhaustion and depersonalization among nurses (H1a) and nurse's aides (H1b).

The* buffering assumption* of the JD-R model [17–19] states that job resources may buffer the impact of verbal aggression on job burnout. Job resources refer to those physical, psychological, social, or organizational aspects that help achieve work goals, reduce job demands, and lessen the associated physiological and psychological costs. As stated above, according to the COR theory [24], verbal aggression is generally perceived to be losses because meeting such demands requires the investment of valued resources, which are viewed as gains [35]. By contrast, the presence of resources in the workplace may interrupt the loss cycle and lead to boosting the motivational process by sustaining the workers in successfully coping with job demands [19, 34]. In this perspective, it is important to understand which resources are useful for dealing with verbal aggression and moderating the development of burnout symptoms.

However, whereas the research is well-developed for most job demands and provides evidence in that direction, as regards verbal aggression, the attention on the variables of the workplace that may buffer its detrimental effects is quite limited [19]. Particularly, the research needs to be expanded in the direction of examining and comparing the roles of different kinds of job resources in buffering the adverse effect of verbal aggression. According to the literature, three types of resources may be available in the workplace: job content resources, social resources, and organizational resources [19]. Rarely in the literature there are studies available that take into consideration, all together, resources from these three levels to test their buffering effects and compare their function in a unique sample. Studies examining all of these resources may advance the literature by indicating the level (job content, social, or organizational levels) to which intervention would be most appropriate [36].

In that direction, the present study includes eight specific resources at the job content, social, and organizational levels. The choice was driven by previous research that recognized the importance of these job characteristics in moderating the effects of the various job demands, including verbal aggression on job burnout, both among the general population and among nursing professionals [15, 35, 37–39].

At the job content level, skill discretion, job autonomy, role clarity, and work meaning were taken into account. According to Karasek [40], skill discretion and autonomy express the extent to which workers are capable of controlling their tasks and general work activities.* Skill discretion* refers to a person's opportunity to use specific job skills in the work process.* Job autonomy* refers to the extent to which a person is autonomous in task-related decisions, such as timing and method control. Broadly speaking, it is plausible that having wide margins of discretion may stimulate workers to exercise creativity in finding successful strategies for managing aggressive patient behaviour, thus lessening exhaustion and depersonalization symptoms caused by exposure to verbal aggression. As regards autonomy, some evidence supports its moderating effect on the relationship between verbal aggression and burnout [15, 38], whereas no studies were found in the literature regarding skill discretion.

*Role clarity* refers to the degree to which the task and the objectives of a job are clearly defined [41]. This job resource has been found to work as a moderator on the relationship between several job demands and workers' outcomes [42, 43]. Even though no studies focus on its role in moderating the relationship between verbal aggression and burnout, it is plausible that role clarity may increase the opportunity to effectively manage the relationship with patients in several ways. For example, workers may be placed in the position to give adequate feedback to patients.

*Work meaning* refers to the degree to which the work is perceived meaningful, important, and constructive [41]. It may work as a buffer of the perceived verbal aggression on burnout by leading the workers to consider the episodes of verbal aggression as learning opportunities for improving care service rather than just as adverse events.

At the social level,* support from colleagues* and* support from supervisors* were considered. Karasek and Theorell [44] defined social support at work as “overall levels of helpful social interaction available on the job from co-workers and supervisors” (page 69). Both supervisors and colleagues may play a role in buffering the burnout symptoms caused by being exposed to patients' verbal aggression by providing both instrumental (i.e., helping workers manage the relationships with patient/relatives) and affective (i.e., giving affective support and not blaming workers for what happens with patients) support. This explanation is consistent with the findings provided by the study from Xanthopoulou et al. [38], which found that social support moderated the detrimental effect of patient harassment on both emotional exhaustion and cynicism in a sample of home care nurses.

At the organizational level, organizational support, fairness, and social utility of the service were considered in the present study.* Organizational support* refers to the degree to which the organization values workers' contributions and the extent it cares about workers' well-being [45]. In a sample of Canadian postal workers, Schat and Kelloway [37] found that organizational support moderated the effects of violence and aggression on emotional well-being and physical health. Based on that, the presence of supportive procedures that help workers when they are victims of aggressive behaviors may help contain the development of burnout.

According to Maslach and Leiter [46],* fairness* reflects organizational justice and can be defined as the extent to which the organization has consistent and equitable rules for all employees. Even if no study specifically explored the moderating role of fairness between verbal aggression and burnout, the literature suggests that it may matter. Elovaino et al. [47] proposed that fairness matters to people because it helps them deal with uncertainty, suggesting that people especially need fair judgments when they are concerned with potential problems associated with social interdependence and socially based identity processes.

*Social utility of the service* refers to the degree to which workers perceive that the organization provides useful and high-quality services for the community [48]. The literature focused poorly on this kind of resource. However, especially in sectors such as health care, in which the link with the community is important, it may play a central role. Indeed, the perception that the service provided by the organization has a positive return for the community may support the workers in keeping a positive self-image, even if some patients show disapproval for their job or the service.

According to the* buffering assumption* [17–19], it is expected that all the resources considered in this study moderate the burnout symptoms among both nurses (H2a) and nurse's aides (H2b). In particular, the relationship between verbal aggression and emotional exhaustion and depersonalization is expected to be stronger when job resources are low rather than when job resources are high.

The literature developed in the framework of the JD-R model regarding the nursing context [17–19], rarely paid specific attention to the various subcategories within the nursing profession, such as nurses and nurse's aides, when the effects of job demands and job resources on psychological health were examined. In particular, previous studies, in most cases, chose to merge these two job categories without verifying the presence of any difference between them despite the fact that nurses and nurse's aides, even if they share the same workplace, significantly differ in educational background, types of tasks they perform, and position in the hierarchy. Nurses have specialized, formal, post-basic education, and they perform more complex tasks such as developing and implementing nursing care plans, maintaining medical records, and administering care to patients. By contrast, nurse's aides have little or no formal training or education and usually assist nurses by carrying out basic, nonspecialized tasks in the care of patients, such as bathing, feeding, and transporting patients under the supervision and the direction of a nurse [49].

Empirical evidence also suggests that merging these groups may obscure the specificity that each category has regarding job stress experience. For example, Seago and Faucett [50] and Morgan et al. [51], using the framework of the Job Demand-Control model (JDC, [40]), found that while nurses fall into the category of active strain (showing high demand and high control), nurse's aides are in the high-strain category (having high demand and low control). Also Fiabane et al. [52] found significantly different distributions on the perception of several work-related psychosocial factors across these two job categories. For these reasons, in the belief that it may be useful to advance the understanding of the phenomenon of job stress in the nursing context, the analyses will be performed separately on the nursing and nursing aides subsamples in the present study to highlight any differences between the two job categories. Due to the exploratory nature of the aim, no expectations can be stated on this point.

The present study may advance the past knowledge on the buffering role of job resources in the demands-burnout relationship because it focuses on some aspects neglected in the previous literature: (a) it considers a wide range of resources (i.e., task level, social level, and organizational level) as possible moderators of the relationship between verbal aggression and burnout and (b) it analyses the buffering mechanism separately within the categories of nurses and nurse's aides.

## 2. Method

Data were collected during a multi-centre intervention-research conducted in four hospitals in Northwest Italy in 2012. Hospital administrations evaluated, endorsed, and authorized the research, allowing researchers to use the data for scientific purposes. Upon approval, department chiefs and nurse coordinators from each ward were asked for authorization to administer the questionnaire to the nurses. An additional ethical approval was not required because no medically invasive diagnostics or procedures were involved to cause psychological or social discomfort for the participants, nor were the patients the subjects of the data collection. However, the research conforms to the provisions of the Declaration of Helsinki in 1995 (as revised in Edinburgh 2000), and all ethical guidelines were followed as required for conducting human research, including adherence to the legal requirements of the study country (Italy).

Participants volunteered for the research and were not asked to sign consent forms, but returning the questionnaire implied consent. The cover sheet clearly explained the research aim, the voluntary nature of participation, the anonymity of the data, and the elaboration of the findings.

The sample consisted of 630 workers: 522 (82.90%) nurses and 108 (17.10%) nurse's aides. The majority were women (81.9%, *n* = 516) aged between 21 and 62 years (*m* = 37.97, sd = 8.76). 57.30% were married or living with partners, 32.20% were single, .90% were divorced, and .60% were widowed.

The average period during which participants had been working in the health-care sector was 13.31 years (sd = 9.02) and ranged from 1 month to 39 years. They were employed in emergency (40.30%) and medical (59.70%) units. Sociodemographic and profession details for nurses and nurse's aides are reported in Table 1.

The data were obtained by means of a self-reported questionnaire that included two sections. The first section collected sociodemographic (gender, age, and marital status) and professional (occupation, units, and years in the health sector) data. The second section included scales aimed at measuring job demand, job resources, and worker outcomes.

### 2.1. Job Demand

*Customer verbal aggression* that was measured by the subscale coming from the Customer-Related Social stressors (CSS) inventory was developed by Dormann and Zapf [20]. The subscale consists of four items (e.g., item: “Patients get angry at us even over minor matters.”) and reports a Cronbach's alpha (*α*) of .92. Responses were given on a four-point scale with a range between 1 (“strongly disagree”) and 4 (“strongly agree”).

### 2.2. Job Resources

Three categories of factors referring to the job content, the social, and the organizational levels were considered. At the job content level, we included four subscales:* work meaning* (5 items, *α* = .761, e.g., item: “Is your work meaningful?”),* role clarity* (3 items, *α* = .72, e.g., item: “Does your work have clear objectives?”),* skill discretion* (5 items, *α* = .61, e.g., item: “My job requires that I learn new things.”), and* job autonomy* (3 items, *α* = .82, e.g., item: “My job allows me to make a lot of decisions on my own.”). The former two were drawn from the Copenhagen Psychosocial Questionnaire by Kristensen et al. [41], and the latter two were taken from the Job Content Questionnaire (JCQ [53]). To measure social resources, two subscales of JCQ [53] were employed. They respectively investigate* support from superiors* (5 items, *α* = .83; e.g., item “My supervisor is helpful in getting the job done.”) and* from peers and colleagues* (6 items, *α* = .82; e.g., item: “People I work with are competent in doing their jobs.”). Three organizational resources were included in the questionnaire. The Organizational Checkup System (OCS [46, 54, 55]) measured fairness (6 items, *α* = .65; e.g., item: “In my organization, job resources are equally distributed.”).* Organizational support* is a scale included in a recent revision of the Job Content Questionnaire (JCQ [53, 56]) (4 items, *α* = .80; e.g., item “My organization really cares about my well-being.”).* Social utility of the service* is a scale drawn from Multidimensional Organizational Health Questionnaire (MOHQ, [48]) and (4 items, *α* = .69; e.g., item: “The organization in which I work provides good service for the community”).

Responses on all subscales were given on a four-point scale with a range between 1 (“strongly disagree”) and 4 (“strongly agree”).

### 2.3. Outcomes

Job burnout was measured thought two subscales from the Italian version of Maslach Burnout Inventory (MBI [57–59]):* emotional exhaustion* (EE, 9 items, e.g., item: “I feel emotionally drained from my work”) and* depersonalization* (DP, 5 items; e.g., item “I feel I treat some patients as if they were impersonal objects”). Both subscales reported a good internal consistency (*α*_EE_ = .82; *α*_DP_ = .77). Responses were given on a seven-point scale (ranging from 0 = “never” to 6 = “every day”).

### 2.4. Control Variables

Gender (0 = male; 1 = female), age, marital status (0 = not living with partner; 1 = living with partner), job seniority, and type of ward (0 = nonacute care ward; 1 = acute care ward) are potential confounders for burnout [57, 58, 60, 61]. In view of that, they were taken into consideration as control variables.

Table 2 reports descriptive statistics (means and standard deviations) and Pearson's correlations for all subscales considered in the study.

All the analyses were performed using SPSS 21. Moderated hierarchical regression analyses were employed to examine the main effect of verbal aggression and of job resources on job burnout, as well as the moderating (buffering) role of job resources on the relationship between verbal aggression and burnout. For each moderated hierarchical regression performed, predictor variables were entered within three successive steps. In the first step, demographical (gender, age, and marital status) and occupational (years in the health sector and type of unit) variables were entered as control variables. In the second step, standardized indexes of verbal aggression and job resources were entered. In the third step, the interaction term, which is the product between verbal aggression and job resource, was entered. When the interaction term showed significant value, the simple slope procedure recommended by Aiken and West [62] was adopted to further examine the pattern of the relationship.

The risk of multicollinearity between independent variables was controlled by standardizing all indexes. Analyses indicated that there were no signs of multicollinearity in any of the regression models. For each independent variable, the tolerance index (1/VIF) never exceeded the score of .70 (cut-off < .20 [63]).

## 3. Results and Discussion

### 3.1. Nurses

Table 3 reports the results of the moderated hierarchical regressions in which emotional exhaustion was entered as a dependent variable. In the third step, all the models reported significant *R* ^2^ and showed a variance explained that ranged from 20% (model 3; JR: skill discretion) to 31% (model 6; JR: support from colleagues). Concerning controlling variables, gender showed a significant association with emotional exhaustion only in model 1 (JR: meaning of the job). The type of unit was found significant in all nine models, indicating that nurses employed in medical units are more prone to develop emotional exhaustion than nurses in the emergency units. Verbal aggression was found to be significant in all models, and its *β* coefficients ranged from .35 to .44.

Regarding the main effect, all the resources we considered, except skill discretion, helped lessen emotional exhaustion. The smallest *β* coefficient was found for meaning of work with .12, and the largest was found for support from colleagues with .35.

The interaction effect between verbal aggression and job resources was found to be significant in models 1, 3, 5, and 7, suggesting that meaning of work (*β* = −.11), skill discretion (*β* = −.11), support from superiors (*β* = −.12), and fairness (*β* = −.11) buffer the effects of verbal aggression on emotional exhaustion.

**Figure 1:**
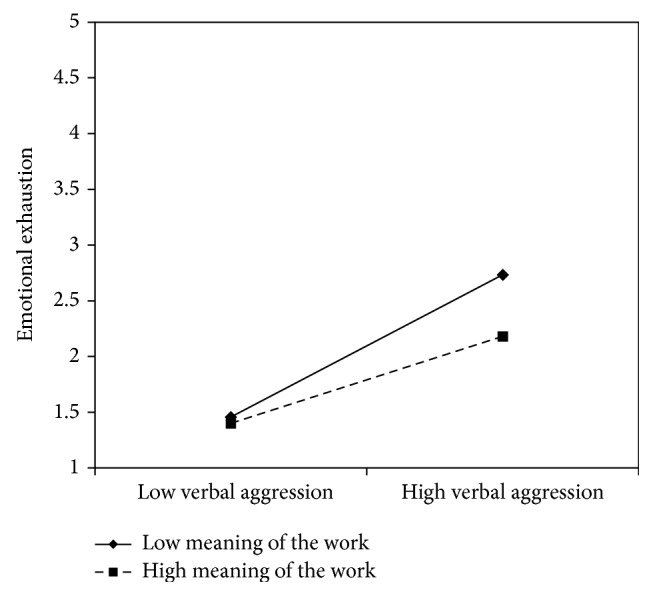
Interaction between verbal aggression and meaning of the work for emotional exhaustion among nurses.

**Figure 2 fig2:**
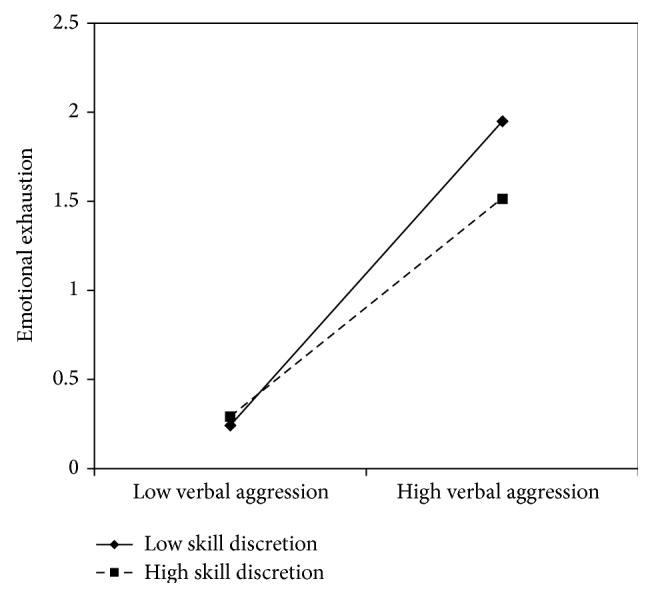
Interaction between verbal aggression and skill discretion for emotional exhaustion among nurses.

**Figure 3 fig3:**
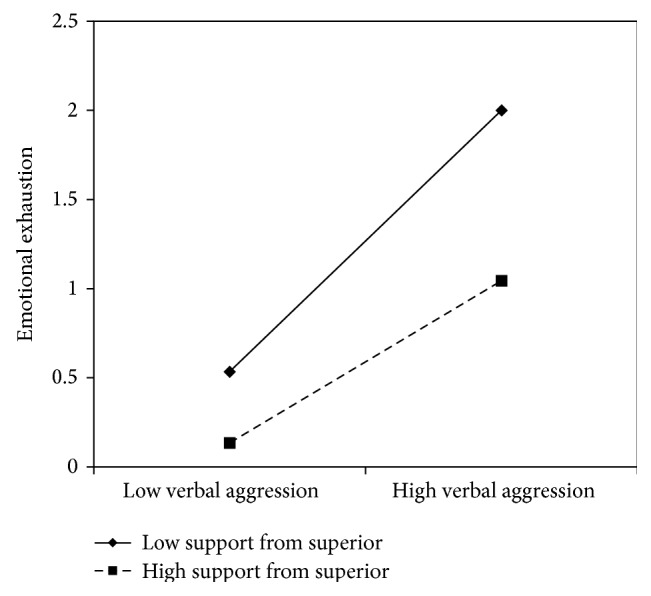
Interaction between verbal aggression and support from superior for emotional exhaustion among nurses.

**Figure 4:**
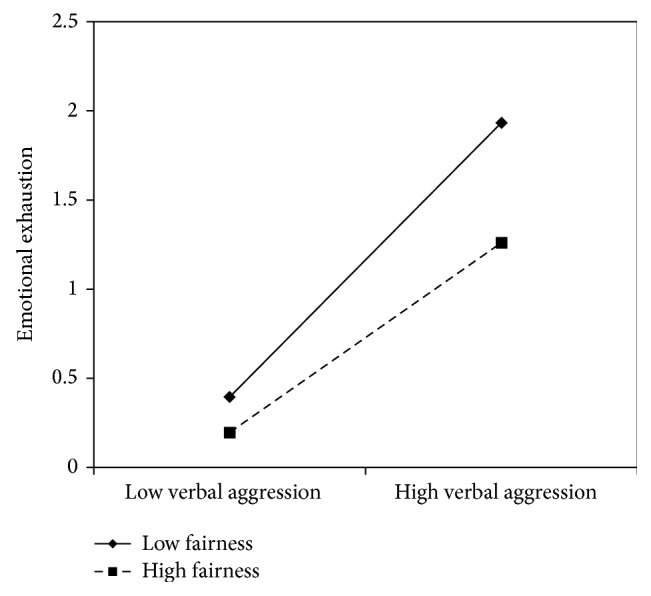
Interaction between verbal aggression and fairness for emotional exhaustion among nurses.

In all these cases, the simple slope analysis (see Figures 1 [Fig fig2] [Fig fig3]–4) showed that when the job resources were high (+1 standard deviation, SD), verbal aggression was positively and significantly related to emotional exhaustion. However, when the job resources were low (−1 SD), the relationship was stronger (*β* = .63, *t* = 7.63, *p* = .00). In particular, for work meaning, the slope at +1 DS showed a *β* of .39 (*t* = 4.67, *p* = .00), whereas at −1 DS, the *β* value reached .63 (*t* = 7.63, *p* = .00). Similarly, the association between verbal aggression and emotional exhaustion was weaker when skill discretion was high (*β* = .61, *t* = 2.65, *p* = .01), rather than when skill discretion was low (*β* = .85, *t* = 5.19, and *p* = .00). Concerning support from superiors, the value of *β* at −1 SD was equal to .73 (*t* = 8.76, *p* = .00), whereas at +1 SD, *β* was equal to .43 (*t* = .43, *p* = .00). Finally, regarding fairness, the value of *β* at −1 SD was equal to .77 (*t* = 8.60, *p* = .00), whereas at +1 SD, *β* was equal to .53 (*t* = 4.83, *p* = .00). Therefore, the slope tests further supported that these resources moderated the effect of verbal aggression in increasing emotional exhaustion in the expected direction.

Table 4 shows the results for depersonalization. Within control variables, gender (in all models) and marital status (in some) were significant. Based on these results, men and people who do not have a partner have more risk of developing depersonalization. Verbal aggression significantly predicted depersonalization in all the models. All resources were found to be significantly related to depersonalization; *β* coefficients indicated that, of these variables, only meaning of work (*β*_DP_ = −.17 to *β*_EE_ = −.12), role clarity (*β*_DP_ = −.19 to *β*_EE_ = −.13), and skill discretion (*β*_DP_ = −.14 to *β*_EE_ = −.07) have a stronger relationship with depersonalization than emotional exhaustion. In the third step, entering the interaction term produced a significant incremental change of *R* ^2^ only for three content-level resources: meaning of work (Δ*R* ^2^ = .03), role clarity (Δ*R* ^2^ = .01), and skill discretion (Δ*R* ^2^ = .02).

**Figure 5:**
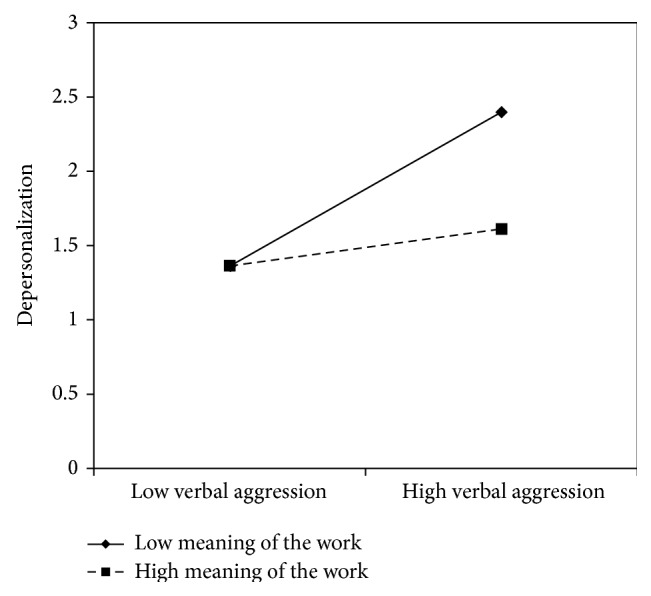
Interaction between verbal aggression and meaning of the work for depersonalization among nurses.

**Figure 6 fig6:**
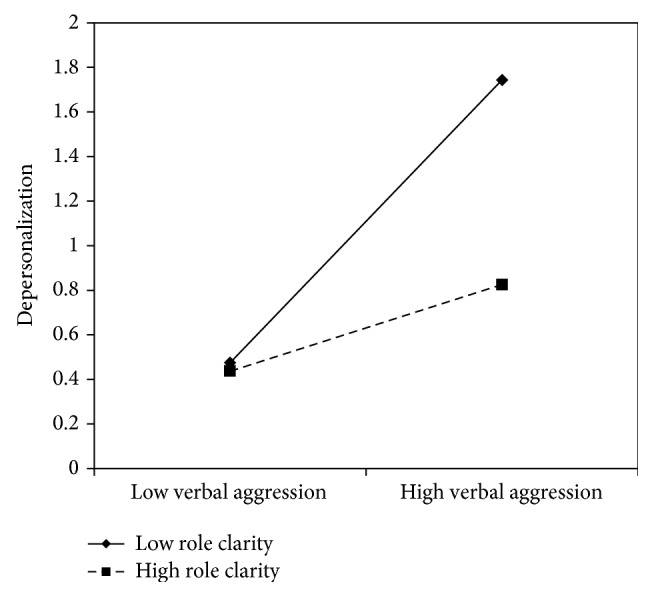
Interaction between verbal aggression and role clarity for depersonalization among nurses.

**Figure 7:**
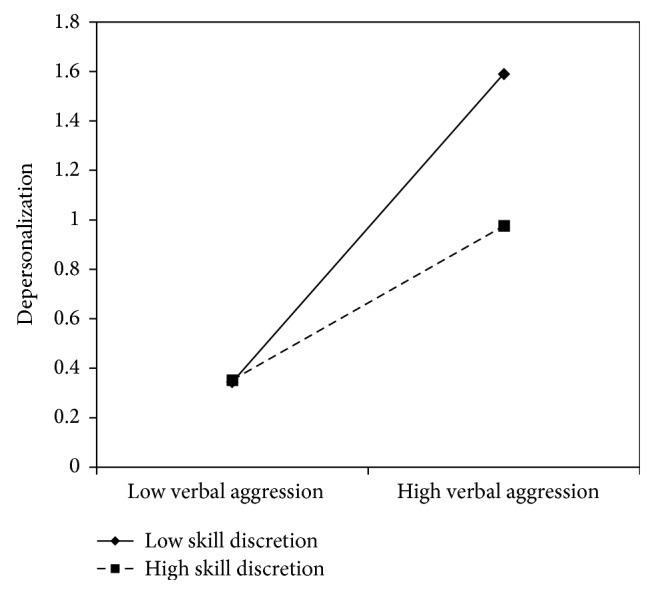
Interaction between verbal aggression and skill discretion for depersonalization among nurses.

Figures 5 [Fig fig6]–7 clearly suggest that meaning of work, role clarity, and skill discretion act as buffers in the relationship between verbal aggression and depersonalization. Further confirmation was provided by the slope test analyses. According to these, when work meaning was high, the association between verbal aggression and depersonalization was not significant (*β* = .12, *t* = 1.61, and *p* = .11), whereas in the case of low work meaning, the relationship between verbal aggression and depersonalization was positive and significant (*β* = .51, *t* = 6.70, *p* = .00). As regards role clarity, the association between verbal aggression and depersonalization was significant in both conditions. However, the relationship was weaker in conditions of high role clarity (*β* = .19, *t* = 2.18, and *p* = .03), rather than in conditions of low role clarity (*β* = .63, *t* = 7.10, *p* = .00). Similarly, for skill discretion, the value of *β* at −1 SD was equal to .62 (*t* = 6.57, *p* = .00), whereas at +1 SD, *β* was equal to .31 (*t* = 3.20, *p* = .00).

These results confirm H1a because, among nurses, verbal aggression was found significantly associated with both emotional exhaustion and depersonalization in all the models. On the other hand, H2a is partially confirmed because the buffer effect of the resource was found in four cases for emotional exhaustion and three cases for depersonalization.

### 3.2. Nurse's Aides

Table 5 shows the results of moderated hierarchical regressions for emotional exhaustion. Control variables showed significant values in none of the cases.

**Figure 8:**
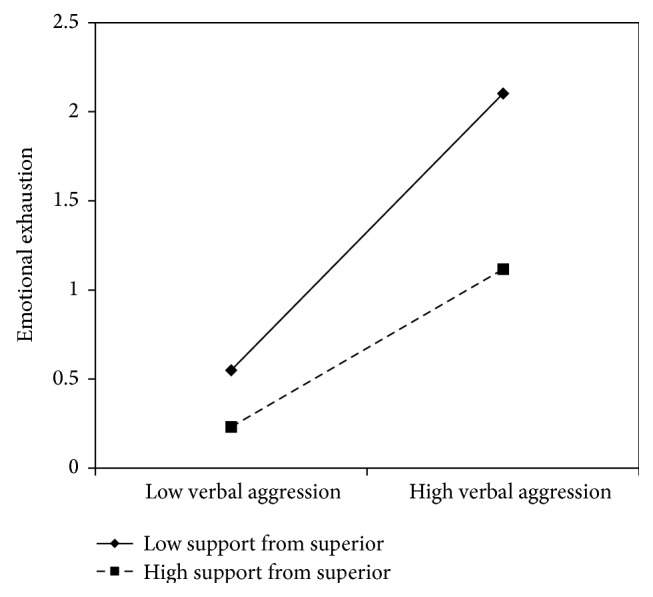
Interaction between verbal aggression and support from superior for emotional exhaustion among nurse's aides.

**Figure 9 fig9:**
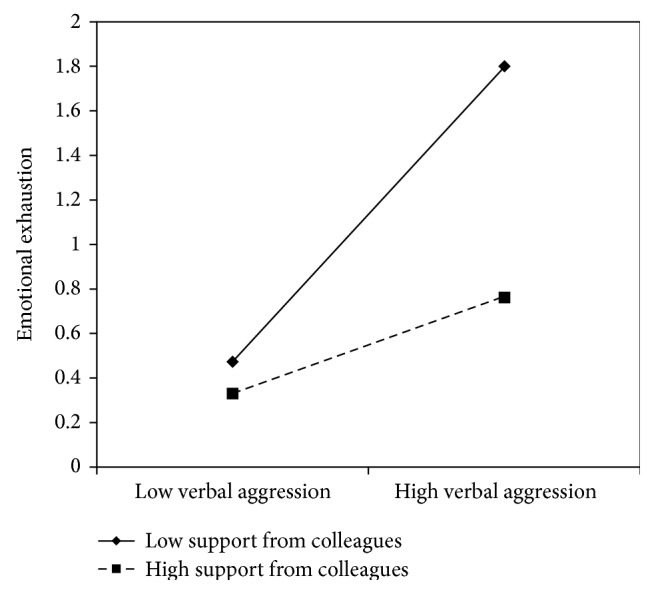
Interaction between verbal aggression and support from colleagues for emotional exhaustion among nurse's aides.

**Figure 10 fig10:**
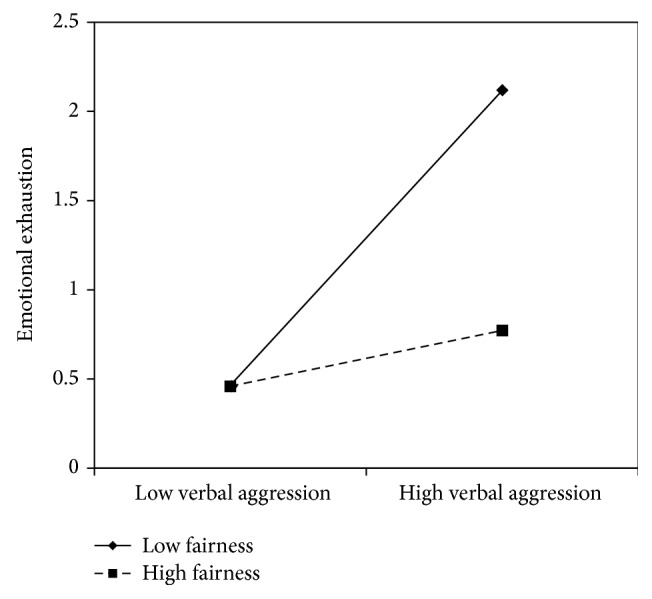
Interaction between verbal aggression and fairness for emotional exhaustion among nurse's aides.

**Figure 11:**
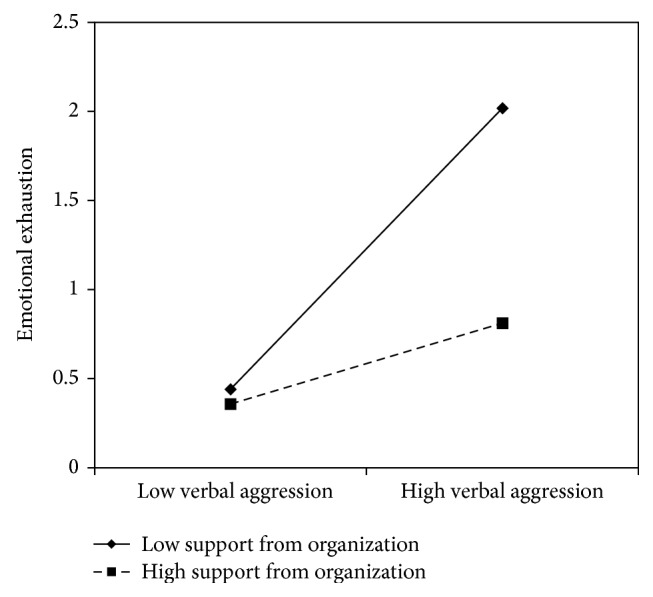
Interaction between verbal aggression and organizational support for emotional exhaustion among nurse's aides.

Concerning verbal aggression, nurse's aides results are similar to the nurses': *β* coefficients in all models showed significant values with the lowest value of .31 and the highest of .50, indicating that verbal aggression positively predicts emotional exhaustion. No content level resources displayed a direct effect on emotional exhaustion. On the contrary, support from superiors (*β* = −.26) and peers (*β* = −.26), fairness (*β* = −.27), organizational support (*β* = −.27), and utility of the service (*β* = −.32) showed a negative significant association with emotional exhaustion. In all of these models, with the exception of the social utility, the interaction terms were also significant. Graphs reported in Figures 8 [Fig fig9] [Fig fig10]–11 indicated the presence of a buffering effect for all these job resources in the relationship between verbal aggression and burnout among nurse's aides.

Further evidence of the moderating role of these job resources was provided by the slope test. As regards support from colleagues, the relationship between verbal aggression and emotional exhaustion was significant at −1 SD (low support from colleagues; *β* = .66, *t* = 4.27, *p* = .00) but not at +1 SD (high support from colleagues; *β* = .21, *t* = 1.09, *p* = .27). Similar results were obtained for fairness (−1 SD: *β* = .82, *t* = 4.84, and *p* = .00; +1 SD: *β* = .15, *t* = .71, and *p* = .47) and organizational support (−1 SD: *β* = .78, *t* = 7.54, and *p* = .00; +1 SD: *β* = .22, *t* = 1.00, and *p* = .31). Concerning support from superiors, the association between verbal aggression and emotional exhaustion was significant in both conditions; however, it was weaker in conditions at +1 SD (*β* = .44, *t* = 2.43, and *p* = .02) rather than at −1 SD (*β* = .77, *t* = 7.40, and *p* = .00).

Table 6 reports results for depersonalization. Gender was significant only in the model in which fairness, organizational support, and social utility were entered. Any other control variables resulted in no significance in the models. Also, in this case, results highlighted that verbal aggression negatively predicted depersonalization (.22 ≤ *β* ≤ .47) in all models.

On the contrary, no resources, except for social utility, showed a direct effect in lessening the depersonalization level among nurse's aides. As highlighted in step three, support from superiors (*β* = −.32), colleagues (*β* = −.38), and the organization (*β* = −.31) and fairness (*β* = −.40) have a role in moderating the negative effect of verbal aggression. As it is possible to see in model 9, social utility is the unique resource that reported both a direct (*β* = −.30) and a moderating (*β* = −.28) effect on depersonalization.

**Figure 12:**
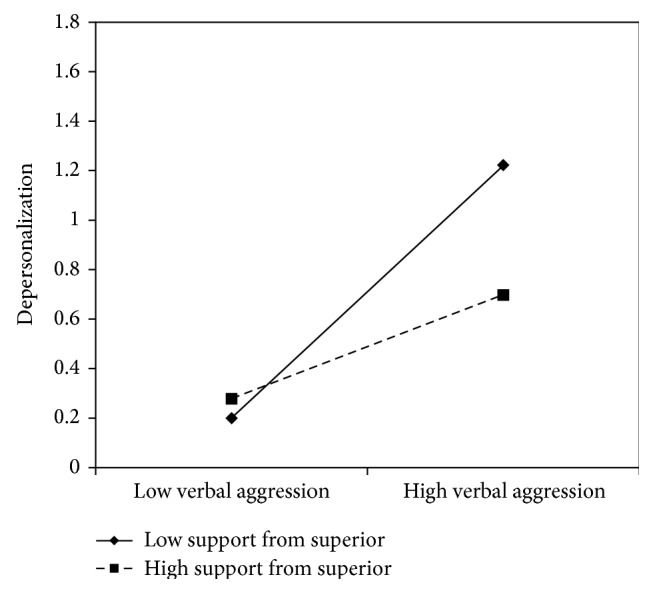
Interaction between verbal aggression and support from superior for depersonalization among nurse's aides.

**Figure 13 fig13:**
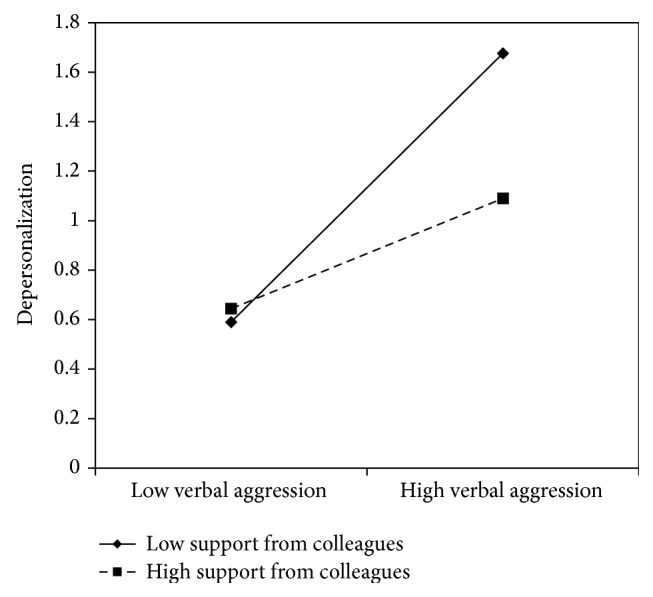
Interaction between verbal aggression and support from colleagues for depersonalization among nurse's aides.

**Figure 14 fig14:**
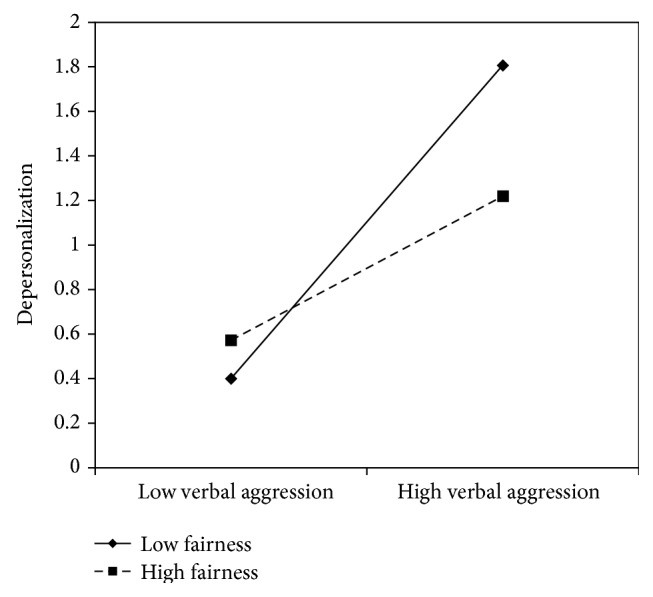
Interaction between verbal aggression and fairness for depersonalization among nurse's aides.

**Figure 15 fig15:**
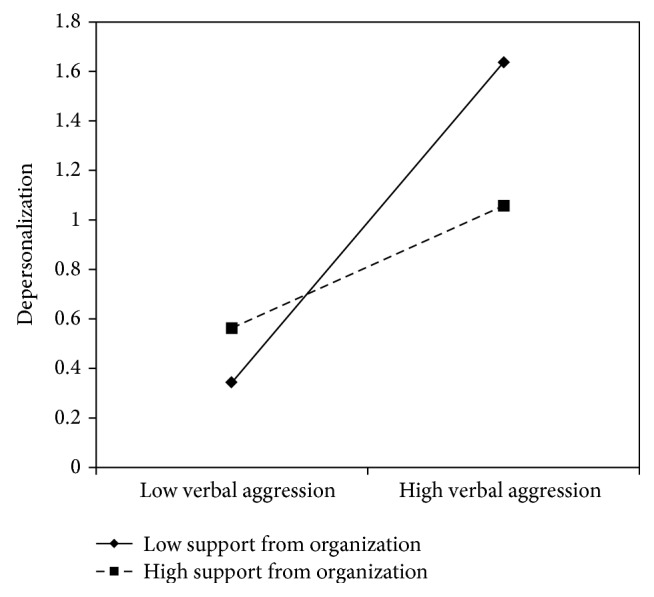
Interaction between verbal aggression and support from organization for depersonalization among nurse's aides.

**Figure 16:**
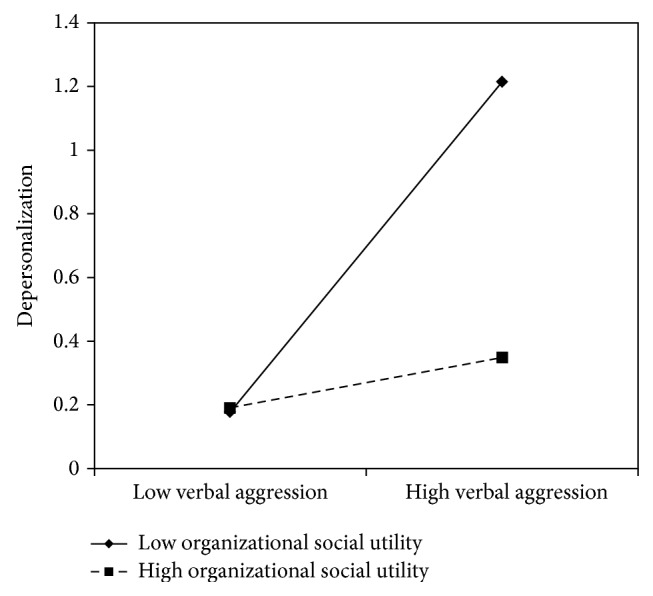
Interaction between verbal aggression and organizational social utility for depersonalization among nurse's aides.

According to the slopes test (see Figures 12 [Fig fig13] [Fig fig14] [Fig fig15]–16), all these resources exercise a buffer effect, thus moderating the negative effect of verbal aggression in increasing nurse's aides depersonalization. Particularly when support from superiors was high, the association between verbal aggression and depersonalization was not significant (*β* = .21, *t* = 1.66, and *p* = .09), whereas in the case of low support from superiors, the association was positive and significant (*β* = .51, *t* = 4.65, and *p* = .00). Also regarding support from colleagues, the relationship between verbal aggression and emotional exhaustion was significant at −1 SD (low support from colleagues; *β* = .66, *t* = 4.27, and *p* = .00) but not at +1 SD (high support from colleagues; *β* = .54, *t* = 5.72, and *p* = .00). As suggested by Figure 14, when fairness was high (+1 DS), verbal aggression was positively and significantly related to depersonalization (*β* = .32, *t* = 4.18, and *p* = .00). However, when fairness was low (−1 SD), the association was considerably stronger (*β* = .70, *t* = 9.08, and *p* = .00). As regards support from the organization, the relationship between verbal aggression and depersonalization was significant at −1 SD (low support; *β* = .59, *t* = 3.25, and *p* = .00) but not at +1 SD (high support; *β* = .12, *t* = .26, and *p* = .79). Similar results were obtained for organizational social utility (−1 SD: *β* = .51, *t* = 3.86, and *p* = .00; +1 SD: *β* = .07, *t* = .42, and *p* = .66).

The results confirm H1b because verbal aggression was significantly associated with both emotional exhaustion and depersonalization in all models carried out among nurse's aides. On the other hand, H2b is partially confirmed because the buffer effect of the resource was found in five cases for emotional exhaustion and in four cases for depersonalization.

## 4. Conclusions

The first aim of the present study was to verify the relationship between verbal aggression and job burnout. The high and significant associations found in both professional groups confirmed the hypothesis that verbal aggression is a predictor of burnout (H1a, H1b). These results suggested that not only in emergency and psychiatry units, as usually pointed out by the literature [64, 65], but also in medical units, dealing with verbal aggression from patients and relatives can be a crucial issue which represents an important emotional demand that contributes to increased burnout levels among nursing staff.

The second aim of the study was to explore whether any job content, social, and organizational level resources are capable of moderating the effect of the exposure to verbal aggression on burnout. The hypothesis that the resources considered moderate the relationship between verbal aggression and the burnout symptoms was only partially confirmed (H2a, H2b). Overall, in 45% of the cases, the cross-product between verbal aggression and the resource was found to be significant. From a general point of view, the findings obtained contribute to enforce the buffering hypothesis of the Job Demands-Resources Model (JD-R, [17–19]), because the interactions found were all in the expected direction. However, it suggests that not all these resources, even if important for reducing burnout (in all cases, job resources showed significant direct and negative associations with emotional exhaustion and in most cases with depersonalization), are useful to cope with verbal aggression. Indeed, results highlight profession-specific patterns in the two occupational subgroups considered.

Considering the job content level among nurses, most of the resources work as moderators of the effect of verbal aggression on burnout. On the contrary, no job content resources work as buffers among nurse's aides. These results could be attributed to the different nature of the work of these two categories. The nurses' work, at the job content level, is richer and more complex than that of nurse's aides and, thus, may offer more resources to successfully deal with the aggressive patients.

These results are also in accordance with those studies which, in the Job Demand Control (JDC) perspective, highlighted that nurses fall into the active strain category, whereas nurse's aides are in the high strain category [50, 51]. However, the present study suggests that for workers who have “poor” job control at the content level, such as nurse's aides, other job resources at the social level and the organizational level may be available and buffer the negative effect of job demand. Indeed, at the social level, among nurse's aides, both forms of support (from peers and superiors) moderated emotional exhaustion and depersonalization. Similarly, at the organization level, most of the resources worked as buffers of verbal aggression among nurse's aides.

On the other hand, it is also interesting to note that among nurses, in most cases, social and organizational resources (with the exception of support from superiors and fairness) did not moderate burnout. These results are difficult to interpret because previous literature rarely pays attention to these aspects. However, an explanation of these results can be found in the Job Characteristic Model by Hackman and Oldham [66]: Aggressiveness may lead workers to develop doubts concerning the worth of their job because patients do not show appreciation for the efforts provided. Richer job characteristics, as in the case of nurses, may allow them to draw energy from the job* per se*, thus making the resources of the job content level available for coping with aggressiveness. This may also be because motivation comes from the work* per se* and not from rewards from patients. This psychological mechanism does not work with nurse's aides, for whom the work* per se* is poorer. Therefore, for them, other aspects of the context such as the social and the organizational environment (i.e., in terms of social and organizational support, opportunity for positive identification in the service provided by the organization, etc.) may be more salient and useful for coping with verbal aggression from patients.

Finally, it is interesting to note that the findings do not support the* matching principle* by De Jonge and Dormann [67]. According to this principle, resources are most likely to moderate the relationship between demands and outcomes if resources, demands, and psychological outcomes all match (e.g., are all at the emotional level). In the present study, it was found that verbal aggression (social stressor) interacted with skill discretion (cognitive resource) in predicting emotional exhaustion (emotional outcome). This finding is in line with some previous studies [38] and suggests that, more than the matching principle, aspects of the work context, including the type of job (e.g., nurses versus nurse's aides), may matter in determining which resources may act as moderators in the relationship between any type of demand and any type of outcome.

Further studies should look more deeply at the difference of the mechanisms that lead to burnout among the two subcategories. Moreover, another suggestion concerns the exploration of the “positive side” of the patient-nurse relationship as a resource able to buffer specifically the “negative side” represented by verbal aggression and exceeding demands [48, 49].

The present study contributes to enlarging empirical evidence developed in the framework of the JD-R model, in particular, by focusing on understudied demands (i.e., verbal aggression) and considering a wide range of resources as its potential moderators.

Moreover, it indicates that more attention should be paid to the study of the stress phenomenon* among* and* across* nurses and nurse's aides because the mechanism that leads to burnout seems to be partially different, especially as regards the functioning of job resources as moderators. From a stress management perspective, the present study suggests that whereas job content level resources should be reinforced to help nurses cope with aggressiveness from patients, as regards nurse's aides, the attention should be focused on the social and organizational levels.

The present study is not without limitations. One concern is that a nonrandomized sampling procedure was used. Even if the sample is quite large, it can limit the generalizability of the results founded. Another important limitation is its cross-sectional design. Therefore, caution must be exercised in the interpretation of the observed associations. It is assumed that job demands and resources are antecedents of burnout, but the opposite could also be true. In fact, elevated rates of burnout could lead workers to develop negative attitudes toward jobs, workplace contexts, and organizations.

## Figures and Tables

**Table 1:** Sociodemographic and professional characteristics of nurses and nurse's aides.

	Nurses		Nurse's aides	
	*n*	%	*n*	%
Gender				
Female	429	82.2	87	80.6
Male	90	17.2	19	17.6
Age				
≤40	288	55.2	33	30.6
>41	234	44.8	75	69.4
Marital status				
Married/living with partner	297	56.9	64	59.3
Single/divorced/widowed	221	42.3	43	39.8
Ward				
Emergency	220	42.1	28	25.9
Medicine	302	57.9	80	74.1
Years in the health sector				
≤15	307	58.8	79	73.1
>16	215	41.2	29	26.9

**Table 2:** Descriptive statistics (means and standard deviations) and Pearson's correlations for all subscales considered in the study.

	M (ds)	(1)	(2)	(3)	(4)	(5)	(6)	(7)	(8)	(9)	(10)	(11)	(12)
(1) Verbal aggression	1.88 (.77)	1											
(2) Meaning of the work	3.38 (.48)	−.11^*∗∗*^	1										
(3) Role clarity	3.34 (.53)	−.11^*∗∗*^	.49^*∗∗*^	1									
(4) Skill discretion	3.36 (.55)	−.03	.57^*∗∗*^	.37^*∗∗*^	1								
(5) Job autonomy	2.68 (.60)	−.08	.36^*∗*^	.30^*∗∗*^	.39^*∗∗*^	1							
(6) Support from superiors	2.84 (.60)	−.13^*∗∗*^	.24^*∗∗*^	.27^*∗∗*^	.14^*∗∗*^	.36^*∗∗*^	1						
(7) Support from colleagues	3.07 (.59)	−.18^*∗∗*^	.30^*∗∗*^	.28^*∗∗*^	.22^*∗∗*^	.30^*∗∗*^	.36^*∗∗*^	1					
(8) Fairness	2.36 (.50)	−.13^*∗∗*^	.15^*∗∗*^	.20^*∗∗*^	**.08**	.27^*∗∗*^	.41^*∗∗*^	.35^*∗∗*^	1				
(9) Support from organization	2.51 (.62)	−.19^*∗∗*^	.18^*∗∗*^	.19^*∗∗*^	.11^*∗∗*^	.41^*∗∗*^	.50^*∗∗*^	.32^*∗∗*^	.57^*∗∗*^	1			
(10) Social utility	2.73 (.54)	−.26^*∗∗*^	.43^*∗∗*^	.39^*∗∗*^	.22^*∗∗*^	.31^*∗∗*^	.36^*∗∗*^	.36^*∗∗*^	.43^*∗∗*^	.43^*∗∗*^	1		
(11) Emotional exhaustion	2.06 (1.28)	.41^*∗∗*^	−.21^*∗∗*^	−.21^*∗∗*^	−.13^*∗∗*^	−.27^*∗∗*^	−.35^*∗∗*^	−.40^*∗∗*^	−.29^*∗∗*^	−.38^*∗∗*^	−.40^*∗∗*^	1	
(12) Depersonalization	1.21 (1.18)	.43^*∗∗*^	−.19^*∗∗*^	−.23^*∗∗*^	−.08^*∗*^	−.12^*∗∗*^	−.23^*∗∗*^	−.20^*∗∗*^	−.18^*∗∗*^	−.21^*∗∗*^	−.33^*∗∗*^	.55^*∗∗*^	1

Note: ^*∗∗*^ < .001; ^*∗*^ < .05.

**Table 3:** Moderated hierarchical regressions to measure main and interaction effects of verbal aggression and job resources on emotional exhaustion among nurses.

Nurses	M1_JR		M2_JR		M3_JR		M4_JR		M5_JR		M6_JR		M7_JR		M8_JR		M9_JR	
	Meaning of the work		Role clarity		Skill discretion		Job autonomy		Support from superior		Support from colleagues		Fairness		Organizational support		Organizational social utility	
**Emotional exhaustion**																		
Step	*β*	*t*	*β*	*t*	*β*	*t*	*β*	*t*	*β*	*t*	*β*	*t*	*β*	*t*	*β*	*t*	*β*	*t*
(1)																		
Gender (1 = female)	.09^*∗*^	1.98^*∗*^	.09	1.87	.09	1.85	.80	1.72	.07	1.48	.08	1.77	.09	1.81	.07	1.51	.07	1.67
Age (1 ≥ 40)	.05	.95	.05	.84	.05	.89	.03	.07	.05	.89	.06	1.16	.04	.67	.40	.70	.05	.85
Marital status (1 = married/partnered = 1)	−.02	−.32	−.01	−.09	−.02	−.40	−.04	−.87	−.05	−1.08	−.04	−.85	−.40	−.82	−.04	−.91	−.17	−.37
Year health sector (1 ≥ 15)	.03	.55	.05	.92	.04	.71	.04	.75	.02	.45	.05	1.05	.04	.75	.01	.34	.03	.55
Type of unit (1 = emergency)	−.14^*∗∗*^	−2.85^*∗∗*^	−.142^*∗∗*^	−2.82^*∗∗*^	−.14^*∗∗*^	−2.73^*∗∗*^	−.12^*∗*^	−2.30^*∗*^	−.10^*∗*^	−2.19^*∗*^	−.14^*∗∗*^	−3.01^*∗∗*^	−.11^*∗*^	−2.17^*∗*^	−.11^*∗*^	−2.35^*∗*^	−.13^*∗∗*^	−2.61^*∗∗*^

(2)																		
Verbal aggression	.42^*∗∗∗*^	8.55^*∗∗∗*^	.43^*∗∗∗*^	8.71^*∗∗∗*^	.44^*∗∗∗*^	9.11^*∗∗∗*^	.41^*∗∗∗*^	8.50^*∗∗∗*^	.36^*∗∗∗*^	7.45^*∗∗∗*^	.35^*∗∗∗*^	7.3^*∗∗∗*^	.40^*∗∗∗*^	7.9^*∗∗∗*^	.37^*∗∗∗*^	7.72^*∗∗∗*^	**.38**	**7.80**
Job resource	−.12^*∗*^	−2.45^*∗*^	−.13^*∗∗*^	2.59^*∗∗*^	−.077	−1.58	−.26^*∗∗∗*^	−5.59^*∗∗∗*^	−.25^*∗∗∗*^	−5.40^*∗∗∗*^	−.35^*∗∗∗*^	−7.51^*∗∗∗*^	−.16^*∗∗*^	−3.46^*∗∗*^	−.29^*∗∗∗*^	−6.08^*∗∗∗*^	−.28^*∗∗∗*^	−6.01^*∗∗∗*^

(3)																		
Verbal aggression × job resource	−.11^*∗*^	−2.25^*∗*^	−.44	−.86	−.11^*∗*^	−2.38^*∗*^	.02	.40	−.12^*∗∗*^	−2.64^*∗∗*^	−.06	−1.25	−.11^*∗*^	−2.30^*∗*^	−.01	−.164	−.07	−1.46

(2) versus (1) Δ*R* ^2^	.21^*∗∗∗*^		.21^*∗∗∗*^		.19^*∗∗*^		.25^*∗∗∗*^		.25^*∗∗∗*^		.31^*∗∗∗*^		.28^*∗∗∗*^		.26^*∗∗∗*^		.26^*∗∗∗*^	
(3) versus (2) Δ*R* ^2^	.01^*∗*^		.00		.01^*∗*^		.00		.01		.00		.09^*∗∗*^		.00		.00	
Adj *R* ^2^	.21^*∗∗∗*^		.21^*∗∗∗*^		.20^*∗∗∗*^		.25^*∗∗∗*^		.27^*∗∗∗*^		.31^*∗∗∗*^		.22^*∗∗∗*^		.25^*∗∗∗*^		.27^*∗∗∗*^	

Note: ^*∗*^.05 ≤ *p* ≤ .011; ^*∗∗*^.01 ≤ *p* ≤ .001;  ^*∗∗∗*^ = .00.

**Table 4:** Moderated hierarchical regressions to measure main and interaction effects of verbal aggression and job resources on depersonalization among nurses.

Nurses	M1_JR		M2_JR		M3_JR		M4_JR		M5_JR		M6_JR		M7_JR		M8_JR		M9_JR	
	Meaning of the work		Role clarity		Skill discretion		Job autonomy		Support from superior		Support from colleagues		Fairness		Organizational support		Organizational social utility	
**Depersonalization**																		
Step	*β*	*t*	*β*	*t*	*β*	*t*	*β*	*t*	*β*	*t*	*β*	*t*	*β*	*t*	*β*	*t*	*β*	*t*
(1)																		
Gender (1 = female)	−.17^*∗∗∗*^	−3.7^*∗∗∗*^	−.16^*∗∗∗*^	−3.50^*∗∗∗*^	−.15^*∗∗*^	−3.23^*∗∗*^	−.17^*∗∗∗*^	−3.66^*∗∗∗*^	−.19^*∗∗∗*^	−3.98^*∗∗∗*^	−.17^*∗∗∗*^	−3.7^*∗∗∗*^	−.18^*∗∗∗*^	−3.71^*∗∗∗*^	−.18^*∗∗∗*^	−3.72^*∗∗∗*^	.19^*∗∗∗*^	−4.08^*∗∗∗*^
Age (1 ≥ 40)	.04	.74	−.00	−.02	.01	.12	−.01	−.18	.02	.27	.01	.25	−.01	−.16	.03	.43	.01	.08
Marital status (1 = married/partnered = 1)	−.10^*∗*^	−2.14^*∗*^	−.07	−1.38	−.09	−1.91	−.11^*∗∗*^	−2.25^*∗∗*^	−.12^*∗*^	−2.43^*∗*^	−.11^*∗*^	−2.12^*∗*^	−.12^*∗*^	−2.37^*∗*^	−.11^*∗*^	−2.17^*∗*^	−.10	−1.78
Year health sector (1 ≥ 15)	−.03	−.63	−.00	−.07	−.02	−.40	−.01	−.21	−.04	−.65	−.01	−.16	−.03	−.63	−.04	−.71	−.02	−.28
Type of unit (1 = emergency)	.08	1.52	.07	1.48	.07	1.36	.07	1.43	.06	1.19	.03	.51	.05	1.05	.06	1.17	.06	1.26

(2)																		
Verbal aggression	.29^*∗∗∗*^	5.90^*∗∗∗*^	.27^*∗∗∗*^	5.62^*∗∗∗*^	.31^*∗∗∗*^	6.27^*∗∗∗*^	.28^*∗∗∗*^	5.53^*∗∗∗*^	.27^*∗∗∗*^	5.24^*∗∗∗*^	.27^*∗∗∗*^	5.13^*∗∗∗*^	.30^*∗∗∗*^	5.72^*∗∗∗*^	.30^*∗∗∗*^	5.98^*∗∗∗*^	.25^*∗∗∗*^	5.06^*∗∗∗*^
Job resource	−.17^*∗∗∗*^	−3.45^*∗∗∗*^	−.19^*∗∗∗*^	−3.74^*∗∗∗*^	−.14^*∗∗*^	−2.75^*∗∗*^	−.18^*∗∗∗*^	−3.71^*∗∗∗*^	−.19^*∗∗∗*^	−3.18^*∗∗∗*^	−.18^*∗∗*^	−3.50^*∗∗*^	−.20^*∗∗∗*^	−4.00^*∗∗∗*^	−.17^*∗∗*^	−3.49^*∗∗*^	−.26^*∗∗∗*^	−5.32^*∗∗∗*^

(3)																		
Verbal aggression × Job resource	−.19^*∗∗∗*^	−3.92^*∗∗∗*^	−.12^*∗*^	−2.41^*∗*^	−.15^*∗∗*^	−3.08^*∗∗*^	−.04	−.73	−.06	−1.29	−.01	−.10	−.01	−.10	.02	.33	−.08	−1.72

(2) versus (1) Δ*R* ^2^	.14^*∗∗∗*^		.15^*∗∗∗*^		.12^*∗∗∗*^		.13^*∗∗∗*^		.13^*∗∗∗*^		.12^*∗∗∗*^		.14^*∗∗∗*^		.13^*∗∗∗*^		.16^*∗∗∗*^	
(3) versus (2) Δ*R* ^2^	.03^*∗∗∗*^		.01^*∗*^		.02^*∗*^		.00		.00		.00		.00		.00		.01	
Adj *R* ^2^	.23^*∗∗∗*^		.21^*∗∗∗*^		.18^*∗∗∗*^		.17^*∗∗∗*^		.18^*∗∗∗*^		.16^*∗∗∗*^		.18^*∗∗∗*^		.18^*∗∗∗*^		.21^*∗∗∗*^	

Note: ^*∗*^.05 ≤ *p* ≤ .011; ^*∗∗*^.01 ≤ *p* ≤ .001;  ^*∗∗∗*^ = .00.

**Table 5:** Moderated hierarchical regressions to measure main and interaction effects of verbal aggression and job resources on emotional exhaustion among nurse's aides.

Nurse's aides	M1_JR		M2_JR		M3_JR		M4_JR		M5_JR		M6_JR		M7_JR		M8_JR		M9_JR	
	Meaning of the work		Role clarity		Skill discretion		Job autonomy		Support from superior		Support from colleagues		Fairness		Organizational support		Organizational social utility	
**Emotional exhaustion**																		
Step	*β*	*t*	*β*	*t*	*β*	*t*	*β*	*t*	*Β*	*t*	*β*	*t*	*β*	*t*	*β*	*t*	*β*	*t*
(1)																		
Gender (1 = female)	.01	.09	.02	.24	.04	.35	.01	.11	−.02	−.21	.01	.14	−.08	−.85	−.04	−.42	−.05	−.54
Age (1 ≥ 40)	.01	.09	.01	.08	.01	.09	.01	.12	−.03	−.27	−.07	−.66	.01	.06	−.06	−.46	.03	.29
Marital status (1 = married/partnered = 1)	−.09	−.86	−.07	−.68	−.10	−1.03	−.10	−.99	−.06	−.62	−.10	−1.06	−.11	−1.23	−.06	−.66	−.07	−.78
Year health sector (1 ≥ 15)	.07	.71	.06	.59	.08	.75	.09	.90	.10	1.04	.12	1.24	.08	91	.05	.50	.11	1.13
Type of unit (1 = emergency)	−.10	−.94	−.09	−.97	−.07	−.70	−.06	−.67	−.01	−.14	.03	.39	.05	.57	−.04	−.50	−.07	−.79

(2)																		
Verbal aggression	.49^*∗∗∗*^	4.61^*∗∗∗*^	.45^*∗∗∗*^	4.69^*∗∗∗*^	.50^*∗∗∗*^	4.39^*∗∗∗*^	.39^*∗∗*^	3.32^*∗∗*^	.38^*∗∗∗*^	4.04^*∗∗∗*^	.31^*∗∗*^	3.10^*∗∗*^	.31^*∗∗*^	3.21^*∗∗*^	.31^*∗∗*^	3.19^*∗∗*^	.32^*∗∗*^	2.99^*∗∗*^
Job resource	−.02	−.23	−.10	−.99	−.16	−1.50	−.16	−1.60	−.26^*∗∗*^	−2.78^*∗∗*^	−.26^*∗∗*^	−2.62^*∗∗*^	−.27^*∗∗*^	−2.90^*∗∗*^	−.27^*∗∗*^	−2.95^*∗∗*^	−.32^*∗∗*^	−3.24^*∗∗*^

(3)																		
Verbal aggression × Job resource	.04	.42	.17	1.80	.14	1.21	−.07	−.68	−.19^*∗*^	−1.94^*∗*^	−.26^*∗∗*^	−2.68^*∗∗*^	−.33^*∗∗*^	−3.53^*∗∗*^	−.27^*∗∗*^	−2.81^*∗∗*^	−.06	−.69

(2) versus (1) Δ*R* ^2^	.21^*∗∗∗*^		.22^*∗∗*^		.22^*∗∗∗*^		.24^*∗∗∗*^		.31^*∗∗∗*^		.31^*∗∗∗*^		.28^*∗∗∗*^		.27^*∗∗∗*^		.30^*∗∗∗*^	
(3) versus (2) Δ*R* ^2^	.00		.03		.01		.00		.03^*∗*^		.05^*∗∗*^		.09^*∗∗*^		.06^*∗∗*^		.00	
Adj *R* ^2^	.19^*∗∗∗*^		.22^*∗∗∗*^		.21^*∗∗∗*^		.21^*∗∗∗*^		.32^*∗∗∗*^		.34^*∗∗∗*^		.35^*∗∗∗*^		.31^*∗∗∗*^		.28^*∗∗∗*^	

Note: ^*∗*^.05 ≤ *p* ≤ .011; ^*∗∗*^.01 ≤ *p* ≤ .001;  ^*∗∗∗*^ = .00.

**Table 6:** Moderated hierarchical regressions to measure main and interaction effects of verbal aggression and job resources on depersonalization among nurse's aides.

Nurse's aides	M1_JR		M2_JR		M3_JR		M4_JR		M5_JR		M6_JR		M7_JR		M8_JR		M9_JR	
	Meaning of the work		Role clarity		Skill discretion		Job autonomy		Support from superior		Support from colleagues		Fairness		Organizational support		Organizational social utility	
**Depersonalization**																		
Step	*β*	*t*	*β*	*t*	*β*	*t*	*β*	*t*	*β*	*t*	*β*	*t*	*β*	*t*	*β*	*t*	*β*	*t*
(1)																		
Gender (1 = female)	−.14	−1.35	−.15	−1.56	−.17	−1.66	−.16	−1.63	−.20	−2.22	−.12	−1.34	−.25^*∗*^	−2.59^*∗*^	−.20^*∗*^	−2.12^*∗*^	−.21^*∗*^	−2.25^*∗*^
Age (1 ≥ 40)	.08	.73	.08	.80	.11	.98	.09	.86	.11	1.09	−.01	−.10	.11	1.07	.08	.74	.09	.93
Marital status (1 = married/partnered = 1)	−.04	−.36	.02	.23	−.01	−.14	−.01	−.08	−.03	−.29	.04	.37	−.45	−.48	−.02	−.25	−.02	−.19
Year health sector (1 ≥ 15)	.04	.33	.01	.02	.02	.23	.04	.37	−.05	−.58	.00	.04	.02	.21	−.07	−.73	.07	.80
Type of unit (1 = emergency)	.02	.21	.02	.18	.03	.28	.02	.17	.08	.93	.13	1.40	.11	1.21	.03	.31	.03	.30

(2)																		
Verbal aggression	.39^*∗∗∗*^	3.72^*∗∗∗*^	.42^*∗∗∗*^	4.41^*∗∗∗*^	.47^*∗∗∗*^	4.15^*∗∗∗*^	.39^*∗∗*^	3.26^*∗∗*^	.35^*∗∗∗*^	3.82^*∗∗∗*^	.28^*∗∗*^	2.91^*∗∗*^	.31^*∗*^	3.16^*∗*^	.34^*∗∗*^	3.32^*∗∗*^	.22^*∗*^	2.11^*∗*^
Job resource	−.09	−.99	−.08	−.85	−.02	−.18	−.03	−.26	−.13	−1.38	−.14	−1.43	−.10	−1.04	−.06	−.65	−.30^*∗∗*^	−3.13^*∗∗*^
(3)																		
Verbal aggression × Job resource	−.10	−1.00	−.15	−1.56	.06	.47	−.09	−.78	−.32^*∗∗*^	−3.32^*∗∗*^	−.38^*∗∗∗*^	−3.85^*∗∗∗*^	−.40^*∗∗∗*^	−4.27^*∗∗∗*^	−.31^*∗∗*^	−3.09^*∗∗*^	−.28^*∗∗*^	−3.02^*∗∗*^

(2) versus (1) Δ*R* ^2^	.20^*∗∗∗*^		.19^*∗∗∗*^		.19^*∗∗∗*^		.19^*∗∗∗*^		.13^*∗∗∗*^		.24^*∗∗∗*^		.19^*∗∗∗*^		.19^*∗∗∗*^		.24^*∗∗∗*^	
(3) versus (2) Δ*R* ^2^	.01		.03		.00		.00		.00		.11^*∗∗∗*^		.14^*∗∗∗*^		.07^*∗∗*^		.07^*∗∗*^	
Adj *R* ^2^	.20^*∗∗∗*^		.21^*∗∗∗*^		.18^*∗∗∗*^		.18^*∗∗∗*^		.32^*∗∗∗*^		.34^*∗∗∗*^		.33^*∗∗∗*^		.26^*∗∗∗*^		.30^*∗∗∗*^	

Note: ^*∗*^.05 ≤ *p* ≤ .011; ^*∗∗*^.01 ≤ *p* ≤ .001; ^*∗∗∗*^ = .00.

## References

[1] NIOSH (National Institute for Occupational Safety and Health) (2002). *Violence: Occupational Hazard in Hospitals*.

[2] OSHA (Occupational Safety and Health Administration) (1996). *Guidelines for Preventing Workplace Violence for Health Care and Social Service Workers*.

[3] Braverman M. (1999). *Preventing Workplace Violence: A Guide for Employers and Practitioners*.

[4] Beech B., Leather P. (2006). Workplace violence in the health care sector: a review of staff training and integration of training evaluation models. *Aggression and Violent Behavior*.

[5] van den Bossche S., Taris T., Houtman I., Smulders P., Kompier M. (2013). Workplace violence and the changing nature of work in Europe: trends and risk groups. *European Journal of Work and Organizational Psychology*.

[6] Camerino D., Estryn-Behar M., Conway P. M., van der Heijden B. I. J. M., Hasselhorn H.-M. (2008). Work-related factors and violence among nursing staff in the European NEXT study: a longitudinal cohort study. *International Journal of Nursing Studies*.

[7] EU-OSHA (European Agency for Safety and Health at Work) (2010). *Workplace Violence and Harassment: A European Picture*.

[8] Arnetz J. E., Hamblin L., Essenmacher L., Upfal M. J., Ager J., Luborsky M. (2015). Understanding patient-to-worker violence in hospitals: a qualitative analysis of documented incident reports. *Journal of Advanced Nursing*.

[9] Duxbury J. A. (2002). An evaluation of staff and patient views of and strategies employed to manage inpatient aggression and violence on one mental health unit: a pluralistic design. *Journal of Psychiatric and Mental Health Nursing*.

[10] Duxbury J. A., Whittington R. (2005). Causes and management of patient aggression and violence: staff and patient perspectives. *Journal of Advanced Nursing*.

[11] Joa T. S., Morken T. (2012). Violence towards personnel in out-of-hours primary care: a cross-sectional study. *Scandinavian Journal of Primary Health Care*.

[12] Gillespie G. L., Gates D. M., Berry P. (2013). Stressful incidents of physical violence against emergency nurses. *The Online Journal of Issues in Nursing*.

[13] Bernaldo-De-Quirós M., Piccini A. T., Gómez M. M., Cerdeira J. C. (2015). Psychological consequences of aggression in pre-hospital emergency care: cross sectional survey. *International Journal of Nursing Studies*.

[14] Magnavita N. (2009). Experience of prevention activities in local health units. Assaults and musculoskeletal disorders. *La Medicina del Lavoro*.

[15] Guglielmetti C., Gilardi S., Accorsi L., Converso D. (2014). La relazione con i pazienti in sanità: quali risorse lavorative per attenuare l'impatto degli stressor sociali?. *Psicologia della Salute*.

[16] Swain N., Gale C. (2014). A communication skills intervention for community healthcare workers reduces perceived patient aggression: a pretest-postest study. *International Journal of Nursing Studies*.

[17] Demerouti E., Nachreiner F., Bakker A. B., Schaufeli W. B. (2001). The job demands-resources model of burnout. *Journal of Applied Psychology*.

[18] Balducci C., Avanzi L., Fraccaroli F. (2014). Emotional demands as a risk factor for mental distress among nurses. *La Medicina del Lavoro*.

[19] Bakker A. B., Demerouti E., Sanz-Vergel A. I. (2014). Burnout and work engagement: the JD–R approach. *Annual Review of Organizational Psychology and Organizational Behavior*.

[20] Dormann C., Zapf D. (2004). Customer-related social stressors and burnout. *Journal of Occupational Health Psychology*.

[21] la Fuente G. A. C.-D., Vargas C., San Luis C., García I., Cañadas G. R., de la Fuente E. I. (2015). Risk factors and prevalence of burnout syndrome in the nursing profession. *International Journal of Nursing Studies*.

[22] Malach-Pines A. (2000). Nurses' burnout: an existential psychodynamic perspective. *Journal of Psychosocial Nursing and Mental Health Services*.

[23] Green D. E., Walkey F. H., Taylor A. J. W. (1991). The three factor structure of the Maslach Burnout Inventory. *Journal of Social Behavior and Personality*.

[24] Westman M., Hobfoll S. E., Chen S., Davidson O. B., Laski S. (2005). Organizational stress through the lens of conservation of resources (COR) theory. *Exploring Interpersonal Dynamics*.

[25] Rogers K. A., Kelloway E. K. (1997). Violence at work: personal and organizational outcomes. *Journal of Occupational Health Psychology*.

[26] LeBlanc M. M., Kelloway E. K. (2002). Predictors and outcomes of workplace violence and aggression. *Journal of Applied Psychology*.

[27] Grandey A. A., Dickter D. N., Sin H.-P. (2004). The customer is not always right: customer aggression and emotion regulation of service employees. *Journal of Organizational Behavior*.

[28] van Emmerik I. J. H., Euwema M. C., Bakker A. B. (2007). Threats of workplace violence and the buffering effect of social support. *Group and Organization Management*.

[29] Mayhew C., Chappell D. (2007). Workplace violence: an overview of patterns of risk and the emotional/stress consequences on targets. *International Journal of Law and Psychiatry*.

[30] Aquino K., Thau S. (2009). Workplace victimization: aggression from the target's perspective. *Annual Review of Psychology*.

[31] Johnson S. J., Holdsworth L., Hoel H., Zapf D. (2013). Customer stressors in service organizations: the impact of age on stress management and burnout. *European Journal of Work and Organizational Psychology*.

[32] Winstanley S., Whittington R. (2002). Anxiety, burnout and coping styles in general hospital staff exposed to workplace aggression: a cyclical model of burnout and vulnerability to aggression. *Work & Stress*.

[33] Hahn S., Müller M., Needham I., Dassen T., Kok G., Halfens R. J. G. (2010). Factors associated with patient and visitor violence experienced by nurses in general hospitals in Switzerland: a cross-sectional survey. *Journal of Clinical Nursing*.

[34] Gascon S., Leiter M. P., Andrés E. (2013). The role of aggressions suffered by healthcare workers as predictors of burnout. *Journal of Clinical Nursing*.

[35] Huynh J. Y., Winefield A. H., Xanthopoulou D. (2013). Social support moderates the impact of demands on burnout and organizational connectedness: a two-wave study of volunteer firefighters. *Journal of Occupational Health Psychology*.

[36] Giga S. I., Cooper C. L., Faragher B. (2003). The development of a framework for a comprehensive approach to stress management interventions at work. *International Journal of Stress Management*.

[37] Schat A. C. H., Kelloway E. K. (2003). Reducing the adverse consequences of workplace aggression and violence: the buffering effects of organizational support. *Journal of Occupational Health Psychology*.

[38] Xanthopoulou D., Bakker A. B., Dollard M. F. (2007). When do job demands particularly predict burnout? The moderating role of job resources. *Journal of Managerial Psychology*.

[39] Heponiemi T., Kouvonen A., Virtanen M., Vänskä J., Elovainio M. (2014). The prospective effects of workplace violence on physicians' job satisfaction and turnover intentions: the buffering effect of job control. *BMC Health Services Research*.

[40] Karasek R. A. (1979). Job demands, job decision latitude, and mental strain: implications for job redesign. *Administrative Science Quarterly*.

[41] Kristensen T. S., Borritz M., Villadsen E., Christensen K. B. (2005). The Copenhagen burnout inventory: a new tool for the assessment of burnout. *Work & Stress*.

[42] Bliese P. D., Castro C. A. (2000). Role clarity, work overload and organizational support: multilevel evidence of the importance of support. *Work & Stress*.

[43] Lang J., Thomas J. L., Bliese P. D., Adler A. B. (2007). Job demands and job performance: the mediating effect of psychological and physical strain and the moderating effect of role clarity. *Journal of Occupational Health Psychology*.

[44] Karasek R. A., Theorell T. (1990). *Healthy Work: Stress Productivity, and the Reconstruction of Working Life*.

[45] Rhoades L., Eisenberger R. (2002). Perceived organizational support: a review of the literature. *Journal of Applied Psychology*.

[46] Maslach C., Leiter M. P. (2008). Early predictors of job burnout and engagement. *Journal of Applied Psychology*.

[47] Elovainio M., van den Bos K., Linna A. (2005). Combined effects of uncertainty and organizational justice on employee health: Testing the uncertainty management model of fairness judgments among Finnish public sector employees. *Social Science & Medicine*.

[48] Avallone F., Paplomatas A., Salute Organizzativa (2005). *Psicologia del benessere nei contesti lavorativi*.

[49] Harris P., Nagy S., Vardaxis N. J., Vardaxis N. (2009). *Mosby's Dictionary of Medicine, Nursing and Health Professions*.

[50] Seago J. A., Faucett J. (1997). Job strain among registered nurses and other hospital workers. *Journal of Nursing Administration*.

[51] Morgan D. G., Semchuk K. M., Stewart N. J., D'Arcy C. (2002). Job strain among staff of rural nursing homes: a comparison of nurses, aides, and activity workers. *Journal of Nursing Administration*.

[52] Fiabane E., Giorgi I., Sguazzin C., Argentero P. (2013). Work engagement and occupational stress in nurses and other healthcare workers: the role of organisational and personal factors. *Journal of Clinical Nursing*.

[53] Karasek R. (1985). *Job Content Instrument Questionnaire and User's Guide, Version 1.1*.

[54] Leiter M. P., Maslach C. (2000). *Organizational Checkup Survey*.

[55] Borgogni L., Galati D., Petitta L., Petitta and Centro Formazione Schweitzer (2005). *Il questionario Checkup organizzativo*.

[56] Cho S. I., Lim S. H., Um K. D. A pilot study for JCQ 2.0 among Korean subway workers.

[57] Maslach C., Jackson S. E. (1986). *Maslach Burnout Inventory*.

[58] Sirigatti S., Stefanile C. (1993). *Adattamento italiano del MBI-Maslach burnout inventory*.

[59] Loera B., Converso D., Viotti S., Federici S. (2014). Evaluating the psychometric properties of the maslach burnout inventory-human services survey (MBI-HSS) among italian nurses: how many factors must a researcher consider?. *PLoS ONE*.

[60] Tummers G. E. R., Landeweerd J. A., van Merode G. G. (2002). The diversity of work: differences, similarities and relationships concerning characteristics of the organisation, the work and psychological work reactions in intensive care and non-intensive care nursing. *International Journal of Nursing Studies*.

[61] Viotti S., Converso D., Loera B. (2012). Job satisfaction, job burnout and their relationships with work' and patients' characteristics: a comparison between intensive care units (ICU) and not-intensive care units (non-ICU). *Giornale Italiano di Medicina del Lavoro ed Ergonomia*.

[62] Aiken L. S., West S. G. (1991). *Multiple Regression: Testing and Interpreting Interactions*.

[63] Field A. (2009). *Discover Statistics Using SPSS*.

[64] Gillespie G. L., Gates D. M., Miller M., Howard P. K. (2012). Emergency department workers' perceptions of security officers' effectiveness during violent events. *Work*.

[65] Gunasekara F. I., Butler S., Cech T. (2011). How do intoxicated patients impact staff in the emergency department? An exploratory study. *The New Zealand Medical Journal*.

[66] Hackman J. R., Oldham G. R. (1980). *Work Redesign*.

[67] de Jonge J., Dormann C. (2003). The DISC model: demand-induced strain compensation mechanisms in job stress. *Occupational Stress in the Service Professions*.

